# Thermophysical properties of the regolith on the lunar far side revealed by the *in situ* temperature probing of the Chang’E-4 mission

**DOI:** 10.1093/nsr/nwac175

**Published:** 2022-08-26

**Authors:** Xiao Xiao, Shuoran Yu, Jun Huang, He Zhang, Youwei Zhang, Long Xiao

**Affiliations:** State Key Laboratory of Geological Processes and Mineral Resources, School of Earth Sciences, Planetary Science Institute, China University of Geosciences, Wuhan 430074, China; State Key Laboratory of Lunar and Planetary Sciences, Macau University of Science and Technology, Macau 999078, China; State Key Laboratory of Geological Processes and Mineral Resources, School of Earth Sciences, Planetary Science Institute, China University of Geosciences, Wuhan 430074, China; Chinese Academy of Sciences Center for Excellence in Comparative Planetology, Hefei 230026, China; China Academy of Space Technology, Beijing 100094, China; China Academy of Space Technology, Beijing 100094, China; State Key Laboratory of Geological Processes and Mineral Resources, School of Earth Sciences, Planetary Science Institute, China University of Geosciences, Wuhan 430074, China

**Keywords:** Chang’E-4, lunar far side, regolith, temperature, thermal conductivity

## Abstract

Temperature probes onboard the Chang’E-4 (CE-4) spacecraft provide the first *in situ* regolith temperature measurements from the far side of the Moon. We present these temperature measurements with a customized thermal model and reveal the particle size of the lunar regolith at the CE-4 landing site to be ∼15 μm on average over depth, which indicates an immature regolith below the surface. In addition, the conductive component of thermal conductivity is measured as ∼1.53 × 10^–3^ W m^–1^ K^–1^ on the surface and ∼8.48 × 10^–3^ W m^–1^ K^–1^ at a depth of 1 m. The average bulk density is ∼471 kg m^–3^ on the surface and ∼824 kg m^–3^ in the upper 30 cm of the lunar regolith. These thermophysical properties provide important additional ‘ground truth’ at the lunar far side, which is critical for the future analysis and interpretation of global temperature observations.

## Teaser

This paper reports the first *in situ* temperature and thermophysical properties analysis of the lunar far side regolith at the CE-4 landing site, performed by a customized thermal model, providing ground truth for future global thermophysical studies of the lunar surface.

## INTRODUCTION

The lunar regolith is a layer of loosely packed rocky grains deposited on the lunar surface, whose physical and chemical properties are important for deciphering geologic history and designing lunar spacecrafts. The thermal conductivity of the lunar regolith, consisting of a conductive component and a temperature-dependent radiative component, is related to the packing style and the size of the solid grains [1] and thus, is a good proxy for this goal. Probing the thermal conductivity of the lunar regolith has drawn a lot of attention since the Apollo era. Early measurements focused on the Apollo regolith samples [2,3], but the experimental data were available only at a few landing sites. Furthermore, the packing style of the regolith samples may have been perturbed during interplanetary transport, which might have affected the reliability of the experimental results. After global thermal mapping by the Lunar Reconnaissance Orbiter (LRO) Diviner radiometer, the thermal conductivity of the lunar regolith was also evaluated via the decrease in surface temperature during the night-time [4,5]. Compared with early laboratory experiments, this method can be implemented without the need to bring samples back from the Moon. Thus, the original packing style of the lunar regolith is not changed, although the measurement accuracy may be affected by the model-dependence and data coverage over time.

The *in situ* temperature probing experiment of the Chang’E-4 (CE-4) mission allows us to track the thermophysical properties of the regolith at the landing site. In this work, we evaluate the thermal conductivity and grain size of the regolith at the CE-4 landing site based on a self-consistent theoretical model.

## TEMPERATURE PROBING EXPERIMENT AND DATA

### The CE-4 lander and temperature probes

**Figure 1.**
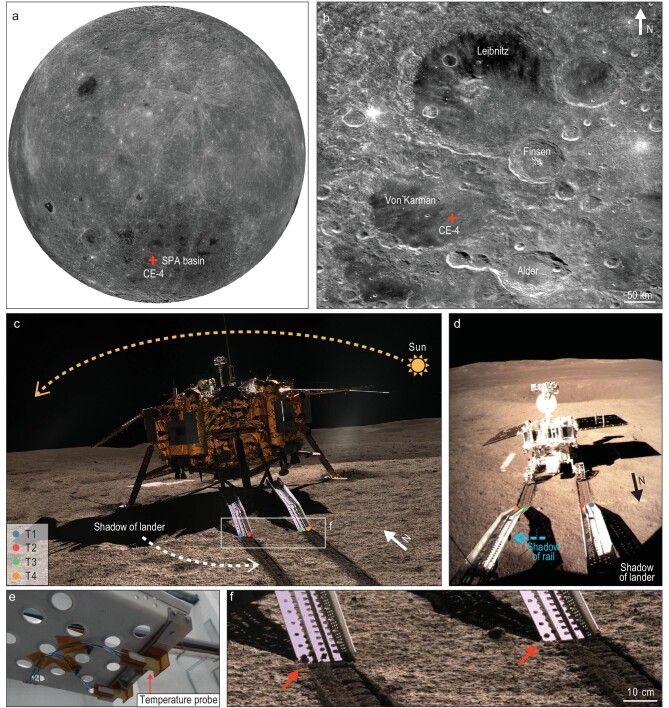
Location of the CE-4 lander and the setting of the temperature probes. (a) The mosaic of the lunar far side obtained by Chang’E-1 (CE-1) charged-coupled device (CCD) camera. The CE-4 landing site (45.4446°S, 177.5991°E) [6] is indicated by the red cross. (b) Regional context of Von Kármán crater. The regolith at the CE-4 landing site (indicated by the red cross) originates from the Finsen crater [17]. The background is the mosaic of CE-1 CCD images. (c) The CE-4 lander on the lunar surface. The yellow dashed arrow indicates the approximate movement direction of the Sun. The white dashed arrow indicates the motion of the lander's shadow along the lunar surface. The colored dots specify the positions of the four temperature probes (T1–T4). The photo was taken by the Panorama Camera on the Yutu-2 rover in the local morning. The rectangle indicates the location of (f). (d) The metallic rails in the local morning. The blue arrow indicates the direction in which the shadow moves across the surface. The positions of the four temperature probes are labeled by the colored dots. (e) The temperature probes installed at the terminals of the metallic rails. (f) Lunar regolith overflow (red arrows) is observed at the end of the rails.

The CE-4 lander landed at 45.4446°S, 177.5991°E [6], on the floor of Von Kármán crater, on 3 January 2019 [7] (Fig. 1a and b). After landing, the Yutu-2 rover was released via two deployed rails orienting

 

towards the south. Four temperature probes (T1–T4 in Fig. 1c and d) beneath the terminals of the rails began to measure the temperature of the local regolith every 900 seconds.

Each temperature probe consists of a low-temperature-type thermistor and a high-temperature-type thermistor, which are sensitive to the temperature of the surrounding materials within 77–223 K and 223–523 K respectively, with an accuracy of ±0.3 K [8]. The temperature probes are powered by solar panels during the day and a radioisotope thermo-electricity generator (RTG) during the night [9], and can provide temperature data throughout a lunation. The thermal radiation from the metallic rails and the lunar lander can also heat up the probed regolith. However, this contribution is nearly negligible during the day, and is just ∼0.16 K during the night [8]. Even so, we still subtracted ∼0.16 K from the measured temperature during the night in the lunar regolith grain size estimation.

The temperature probes were installed at the terminals of the metallic rails by the polyimide holders (Fig. 1e), whose thermal resistance (∼5000 K W^–1^) is high enough to impede the thermal influence of the metallic rails [8]. The thermal conductivities of metals and polyimide are typically hundreds of W m^–1^ K^–1^ [10] and ∼0.12 W m^–1^ K^–1^ [11] respectively, far greater than the thermal conductivity of the lunar regolith. As we prove in Supplementary Discussion 1, these two overlapping layers do not perturb thermal conductivity on the lunar surface or, therefore, heat conduction in the topmost lunar regolith.

In order to acquire the temperature of the local regolith, the temperature probes must have contact with the regolith sufficiently. Figure 1f shows the terminals of two metallic rails on the lunar regolith. We observe the overflow of regolith in the lowest holes, which implies that the metallic rails have compressed the underlying regolith significantly. Correspondingly, the temperature probes would have been surrounded by the local regolith.

### Temperature data

**Figure 2.**
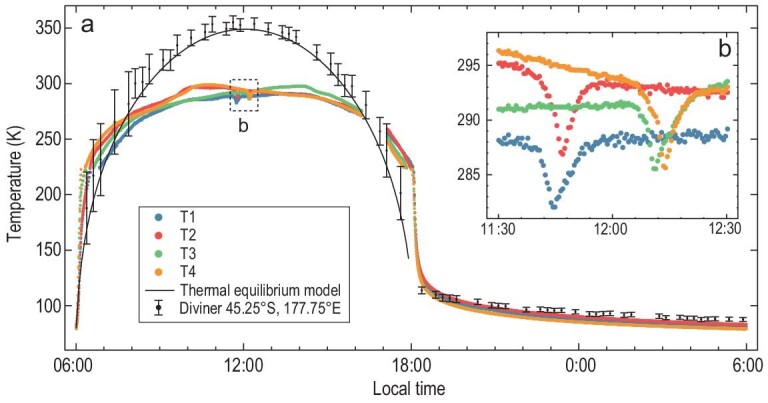
The temperature variation at the CE-4 landing site obtained during the third lunar day after landing. (a) The colored scatter plots represent the temperatures measured by the temperature probes on the CE-4 lander. The black curve represents the temperature during the day, simulated by the thermal equilibrium model with the assumption of direct solar illumination (see Supplementary Methods). The black scatter plots are Diviner bolometric temperatures for an area (45.25°S, 177.75°E, 0.5° in width) containing the CE-4 landing site and the vertical bars represent estimated error bounds [22]. (b) The temperature measured near the lunar noon of the third lunar day after landing.

We obtained the temperature data collected between the second and fourth month after the landing. As an example, Fig. 2a shows the temperature data of T1–T4 between 9:28:08, 27 February 2019 and 21:31:57, 28 March 2019 (UTC time), i.e. the third lunar day after landing. In this study, we only use the temperature measurement from T2 during the third lunar day for analysis, as it has the best data quality. Firstly, the temperature data of the other three probes are discontinuous in time at the threshold temperature (∼223 K) alternating the thermistors (see Supplementary Fig. 6a). Secondly, the temperature data of probe T2 in the other lunar nights are discontinuous (see Supplementary Fig. 6b).

### Buried depth of the temperature probes

Although the temperature probes have direct contact with the local regolith, there was no equipment to tell how deeply they were buried. Here we determine the buried depth of the four temperature probes by the time when the temperature begins to increase in local morning. If the temperature probes have contact with the very topmost regolith, the sunrise-caused temperature increase should be simultaneous with the sunrise itself. Otherwise, there should be a delay between them (see Supplementary Fig. 9). Following our statistics (see Supplementary Table 2), the measured temperature begins to increase at 09:13, 27 February 2019 for all four temperature probes, nearly simultaneous with the local sunrise at 09:15, 27 February 2019, determined by equaling solar altitude angle and terrain occlusion angle (see Supplementary Fig. 7) [12], considering the 900-second intervals of the temperature measurement sampling. Therefore, we conclude that the buried depths of all four temperature probes are <1 cm, and the measured temperature should characterize the thermal conduction of the very surficial regolith.

During the CE-4 landing, the rocket exhaust may have blown away the original topmost regolith below the nozzle. According to erosion depth modeling [13], the surface erosion depth at the position of the temperature probes is only ∼0.5 mm, which is nearly negligible (see Supplementary Discussion 2 for details).

### Influences of shadows and ambient scattering

During the day, the temperature of the lunar regolith is dominated by the absorbed thermal radiation from the ambiance. As in Fig. 1c and d, the shadows of the metallic rails and lunar lander may complicate the time variation of the ambient radiation. As a reference, we evaluate the surface temperature of daytime by the thermal equilibrium model, with the assumption of direct solar illumination (see Supplementary Methods). As in Fig. 2a, the measured temperatures are higher than the theoretical values before 07:00–08:00 and after 16:00, but are lower than the theoretical values between them. The greatest difference appears at the lunar noon with a magnitude of ∼55–60 K. Besides, the climax in temperature appears at ∼10:00 for the probes T2 and T4, but at ∼14:00 for the probes T1 and T3, deviating significantly from the climax of the theoretical temperature curve, which lies at ∼12:00. The special behavior of the measured temperature is mainly related to the shadows of the rails and the lander itself. For a more detailed discussion, refer to Supplementary Discussion 3.

By comparing the measured temperatures with the theoretical values predicted by the thermal equilibrium model assuming direct solar illumination, the higher measured temperature before 7:00–8:00 and after 16:00 implies a strong enhancement of external radiation, which can only be induced by the scattered sunshine on the down-sun side of the lander. Nevertheless, the shadow of the lander moves counter-clockwise when viewed from the top of the lander (Fig. 1c). Accounting for this factor, the shading effect of the lander, which dominates the solar radiation and the total intensity of the scattered sunshine on the down-sun side of the lander, tends to be enhanced over time in the morning, reaches the strongest level at the lunar noon, and tends to weaken over time in the afternoon. Hence, the enhancement of the scattered sunshine can only appear near the two end sides of daytime. At other times, the shadow of the lander may suppress the intensity of the solar radiation reaching the down-sun side of the lander, as well as the total intensity of the scattered sunshine. Therefore, the surface temperatures remain lower than the theoretical values.

The shadow of the metallic rails can also bias the measured temperature. During 7:00/8:00–16:00, T2 and T4 detected the maximum temperatures 297.0 and 298.5 K respectively at ∼10:00, whereas the probes T1 and T4 detected the maximum temperatures 290.9 and 297.5 K respectively at ∼14:00. All these climaxes deviate significantly from the maximum theoretical value 348.4 K, appearing at ∼12:00. This inconsistency can be explained by the shadow of the metallic rails. In the morning, the shadow of either metallic rail falls onto the right side of the rail itself (Fig. 1c). In this case, T1 and T3 are totally shaded, whereas T2 and T4 are less shaded. As a consequence, the increase of temperature at T2 and T4 is more rapid than that at T1 and T3. Also, the shadow of the metallic rail always moves towards the east and therefore the shading effect at T2 and T4 is strengthened over time, which further decelerates the increase of the surface temperature. Finally, these two effects result in the maximum temperature appearing prior to the local noon at T2 and T4. In the afternoon, the shadow falls onto the left side of the rail. In contrast to the morning case, T2 and T4 are totally shaded, whereas T1 and T3 are less shaded. Hence, the temperatures at T2 and T4 tend to decrease, but the temperatures at T1 and T3 can keep increasing until ∼14:00 local time.

We observe four sudden temperature drops of 5–8 K at 11:30–12:30 local time (Fig. 2b). These temperature drops appear in the order T1–T4 over time, consistent with the order in which the temperature probes are covered by the lander's passing shadow (Fig. 1c). This feature likely characterizes the strong shading effect of the lander at local noon.

## RESULTS

### Grain size at the CE-4 landing site

We express the bulk density and thermal conductivity of the lunar regolith in terms of grain size (see Supplementary Methods) and couple them with a temperature model based on the heat conduction equation. Here the regolith grains are considered to be equal-sized and the grain size is also assumed to be constant over depth. In particular, we consider the influence of rail-induced surface pressure on the filling factor profile of the lunar regolith, which can modify its bulk density and thermal conductivity for all depths. Then, the grain size is estimated by fitting the modeled surface temperature with the measured surface temperature.

During the day, the temperature of the topmost regolith is governed by the absorbed external radiation. Accounting for the shading effect, we calculate an effective radiation flux based on the measured surface temperature (see Supplementary Methods). In this way, the modeled temperatures of daytime are close to the measured temperatures during the day (see Supplementary Fig. 8).

**Figure 3.**
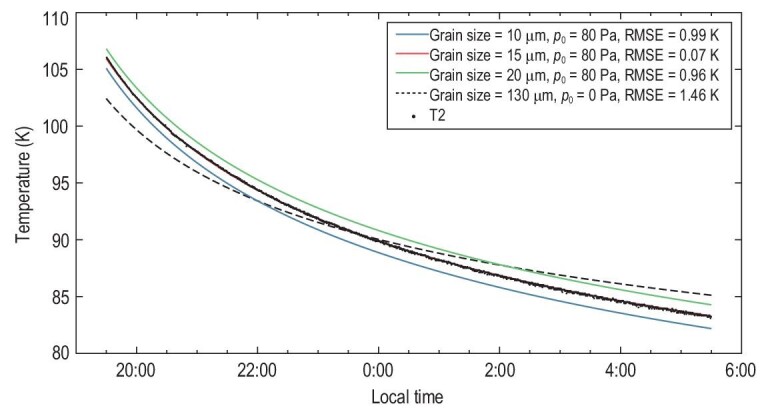
Comparisons between the modeled temperatures of various grain sizes with surface pressure and the measured temperatures of the probe T2 during the night. The colored curves specify the decrease of surface temperatures modeled for the grain sizes 10 μm, 15 μm and 20 μm with a surface pressure of 80 Pa, and the black dashed curve for the grain size 130 μm without surface pressure, whereas the black dots specify the temperatures measured by the probe T2. The best fit between the modeled temperatures and the measured temperatures is achieved with a grain size of ∼15 μm, with a surface pressure of 80 Pa.

During the night, the decrease of surface temperature is sensitive to the variation of the grain size and the surface pressure (Fig. 3). By varying these two parameters, the best fit between the modeled temperatures and the measured temperatures is achieved with a grain size of ∼15 μm and a surface pressure of ∼80 Pa. Here, we use only the data between 19:30 and 05:30 to avoid the complex topographic influences near sunset and sunrise [4].

In our thermal model, we assume the regolith radiates towards a free space. However, the thermal radiation from the metallic rails can slow down the radiative cooling of the regolith below. As in Supplementary Discussion 4, we prove that the thermal radiation from the metallic rails tends to be 10% of the thermal radiation from the regolith during the ∼0.4 lunar hours (i.e. ∼11 terrestrial hours) after sunset. Hence, the assumption of free space is still applicable here.

### Physical meaning of the estimated grain size

The size of regolith grains can be described by different indicators, such as mass-weighted grain size and number-weighted grain size. In early experiments for the Apollo regolith samples, the regolith grains were investigated based on the sieving method. This method can yield the mass fractions of the solid grains in different sizes and thus the mass-weighted grain size was adopted as the major size indicator [14]. In our model (see Supplementary Methods), the grain size is related directly to the geometry of regolith grains, which is dominated by the solid grains with the highest number fraction. Hence, the estimated grain size should have the same significance as the number-weighted grain size.

We also assumed the grain size to be constant over depth, but this assumption would be in discrepancy with the varying grain size over depth in the Apollo drilled samples. Note that this is a trade-off option when considering the fitting scheme. Constraining the variation of grain size over depth requires two free variables at least, which in turn results in the multiple-solution problem in the fitting. Due to the lack of samples from the CE-4 landing site, it is still hard to set a reference to constrain the solution. Even so, the estimated grain size is still meaningful. As indicated in Supplementary Methods, the grain size can affect the thermophysical properties of the lunar regolith at all depths. Correspondingly, the estimated grain size likely characterizes an average case for the grain sizes at all depths.

### Thermophysical properties of the regolith at the CE-4 landing site

**Figure 4.**
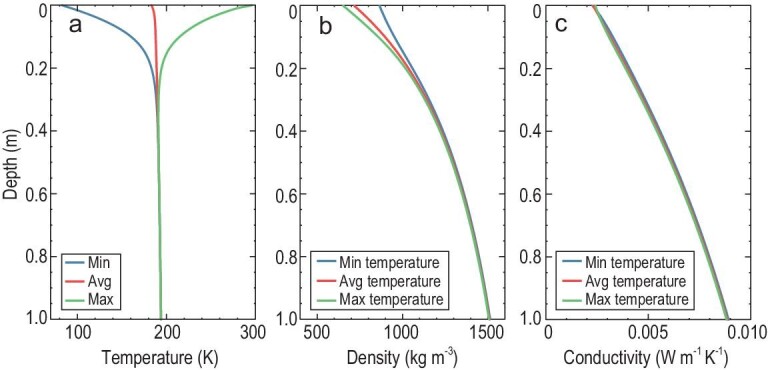
The profiles of temperature, bulk density and conductive component of thermal conductivity at the CE-4 landing site with a surface pressure of 80 Pa. (a) The minimum, average and maximum temperature profile from the surface to a depth of 1 m. (b) The bulk density profile from the surface to a depth of 1 m, corresponding to the minimum, average and maximum temperatures in Fig. 4a. (c) The conductive component of the thermal conductivity profile from the surface to a depth of 1 m, corresponding to the minimum, average and maximum temperatures in Fig. 4a.

Following the estimated grain size, we calculate the profiles of temperature, bulk density and the conductive component of thermal conductivity from the surface to a depth of 1 m at the CE-4 landing site (Fig. 4). With the load of the metallic rails on the lunar surface (i.e. *p_0_* = 80 Pa), the bulk density is 651–865 kg m^–3^ and 717 kg m^–3^ on average on the surface. It increases rapidly over the topmost ∼0.3 m, and finally converges towards 1840 kg m^–3^ at a depth of 5 m. In particular, the surface bulk density presents a diurnal variation of ∼200 kg m^–3^ relating to the temperature dependence of adhesive bonding force [15], and consequently a time-varying turnover pressure marking the transition of packing style (see Supplementary Methods). The conductive component of the thermal conductivity is ∼2.27–2.42 × 10^–3^ W m^–1^ K^–1^ and ∼2.30 × 10^–3^ W m^–1^ K^–1^ on average on the surface. It increases rapidly over the topmost ∼0.3 m and finally converges towards 8.86 × 10^–3^ W m^–1^ K^–1^ at a depth of 1 m. The diurnal variation of the conductive component is also related to the temperature dependence of adhesive bonding force [15].

**Figure 5.**
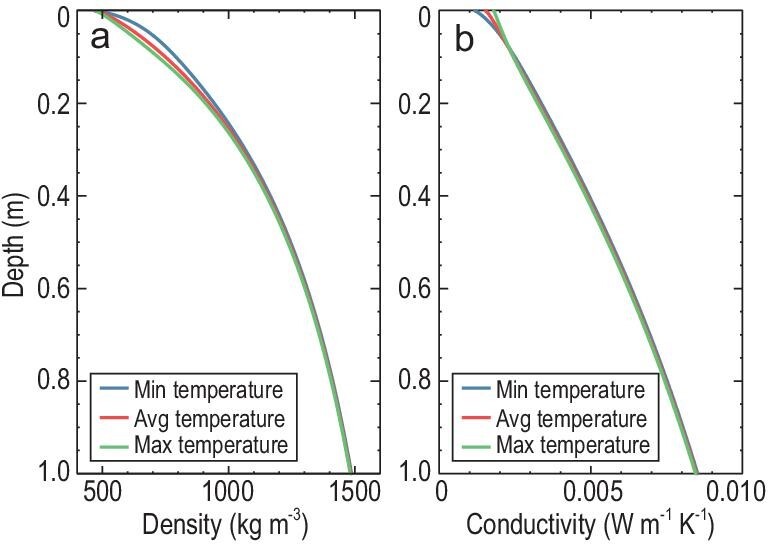
The profiles of bulk density and conductive component of thermal conductivity at the CE-4 landing site without surface pressure. (a) The bulk density profile from the surface to a depth of 1 m, corresponding to the minimum, average and maximum temperatures in Fig. 4a. (b) The conductive component of thermal conductivity from the surface to a depth of 1 m, corresponding to the minimum, average and maximum temperatures in Fig. 4a.

By removing the load on the lunar surface, i.e. *p_0_* = 0 Pa, the bulk density is ∼471 kg m^–3^ on the surface and finally converges upon ∼1838 kg m^–3^ at a depth of 5 m, and the average bulk density in the upper 30 cm of the lunar regolith is 824 kg m^–3^. The thermal conductivity is ∼1.17–1.79 × 10^–3^ W m^–1^ K^–1^ and ∼1.53 × 10^–3^ W m^–1^ K^–1^ on average on the surface, and finally towards ∼8.48 × 10^–3^ W m^–1^ K^–1^ at a depth of 1 m (Fig. 5).

## DISCUSSIONS

### Immature regolith layer below the lunar surface

The regolith grains are crushed and comminuted as a result of exposure to micrometeorite impacts, cosmic rays and solar winds [16]. As a consequence, the regolith grains are coarse in the young regions, but tend to be finer and finer as they increase in age. The lunar regolith at the CE-4 landing site evolved mostly from the ejecta of the Finsen crater, i.e. an impact crater formed ∼3.6 Gyr ago [17]. However, the estimated grain size is greater than the number-weighted grain size of Chang’E-5 (CE-5) regolith samples (3.5–4.0 μm) [18,19], which were collected from the lunar surface and have an age of ∼2.0 Ga [20].

As we mentioned above, the estimated grain size represents the average grain size at all depths. One plausible explanation is that the regolith below the surface of CE-4 landing site is abnormally immature. Note that this speculation is also favored by the spectral observation at the CE-3 landing site, i.e. the exposed regolith is less mature than the removed topmost regolith after being subjected to rocket exhaust during the landing [21]. If this speculation is correct, space weathering may be drastic only on the lunar surface, but tends to be much weaker in the lunar subsurface.

In order to confirm the speculation above, we also exploit our fitting scheme to investigate the grain size at the CE-5 landing site (see Supplementary Discussion 5). By using the Diviner bolometric temperatures at 43.25°N, 51.75°W within ±0.25° [22] around the CE-5 landing site (43.06°N, 51.92°W) [23], the estimation yields a grain size of ∼200 μm, or ∼110–330 μm, accounting for the error of the data. The result is greater than the estimated grain size at the CE-4 landing site, which agrees well with the young age of the CE-5 landing site and the relatively older age of the CE-4 landing site. However, the number-weighted grain size of CE-5 samples is far smaller than the result here. This inconsistency may also imply that the regolith below the surface of the CE-5 landing site is much coarser than that on the surface. In addition, the thermal conductivity on the surface of the CE-5 landing site is 0.56 × 10^–3^ W m^–1^ K^–1^, or ∼0.49–0.69 × 10^–3^ W m^–1^ K^–1^, accounting for the error of the data. This conductivity component is similar to the value in the young regions, i.e. ∼0.5–0.7 × 10^−3^ W m^–1^ K^–1^ [5], which is consistent with the young units (∼2.0 Ga) at the CE-5 landing site [20].

### Low bulk density on the surface

Following previous work on the Apollo drilled samples, the density of the lunar regolith is ∼1300 kg m^–3^ on the surface, increases rapidly over the topmost tens of centimeters and then converges 1920 kg m^–3^ below [16]. In our results, the bulk density of the deep regolith is estimated as 1838 kg m^–3^, nearly consistent with the earlier recommended value for the deep regolith. Nevertheless, the topmost regolith shows a low bulk density of ∼471 kg m^–3^ in the load-free case, far lower than the earlier recommended value.

In fact, this low bulk density on the surface is related to the low filling factor of ∼0.15 prescribed for the uncompressed topmost regolith, characterizing the random ballistic packing (RBP) [24]. We note that the earlier model for the density profile was obtained by fitting the average densities of several regolith sections, sampled by core tubes, over depth [16]. The density of the topmost regolith was actually extrapolated from the fitted curve, but was never measured directly. In contrast, the filling factor of RBP used by us is verified by theoretical and experimental studies [25] and is thus a more reliable option for the uncompressed topmost regolith. On the other hand, using the filling factor of RBP and the turnover pressure, i.e. a pressure marking the transition of packing style, defined in Supplementary Methods, can well reproduce the filling factor over the topmost 15 cm, as recommended by the experiments on Apollo drilled samples [24].

According to the data of the Yutu-2 Lunar Penetrating Radar (LPR), the bulk density of surface regolith (<30 cm) at the CE-4 landing region was estimated to be 1310 ± 200 kg m^–3^ [26], which is slightly greater than the value of 824 kg m^–3^ derived from our estimation. The bulk density estimated by the Yutu-2 LPR data approaches a constant value (1900 ± 80 kg m^–3^) below 5.8 m [27], very close to the value of 1838 kg m^–3^ derived from the temperature. However, we note that the density estimated by radar observations was conducted based on the measured dielectric constant of lunar regolith, whose relationship with bulk density and TiO_2_ content is still largely uncertain [28,29]. Hence, it is unknown whether this method is suitable for the estimation of bulk density.

### Thermal conductivity of the topmost regolith

In previous work, the Diviner data were used to describe the diurnal and seasonal thermophysical properties (e.g. thermal conductivity: Table 1) of the regolith at very shallow depths (∼10–100 cm) [30]. However, the thermal conductivity of the topmost regolith layer (within ∼1 cm) is difficult to constrain due to its relatively small amount of mass [31]. On the other hand, the thermal conductivity of the topmost regolith and its transitional rate over depth are sensitive to the decrease of surface temperature during the night, and the effects of these two parameters on the surface temperature decrease during the night are strongly coupled [4,5]. Hence, earlier works estimated one parameter by fixing the other to the typical value. In this work, these two parameters are expressed in terms of the grain size (see Supplementary Methods). Correspondingly, our estimation is not affected by the parameter coupling and thus is more reliable.

**Table 1.** The conductive component of thermal conductivity for the topmost regolith.

Data sources	Latitude	${k}_s$ (W m^–1^ K^–1^)
Apollo 12 samples [2]	3°	1.2–1.6 × 10^–3^
Apollo 15 samples [3]	26°	∼0.8 × 10^–3^
Apollo 15 *in situ* [33]	26°	0.9–1.6 × 10^–3^
Apollo 17 *in situ* [34]	20°	0.9–1.5 × 10^–3^
*Model* of Vasavada *et al*. [30]	Equatorial	0.6 × 10^–3^
*Model* of Yu and Fa^a^ [5]	Older regions	0.9–1.2 × 10^–3^
	Younger regions	0.5–0.7 × 10^–3^
*Model* of Hayne *et al*. [4]	Equatorial	0.74 × 10^–3^
This study, CE-4 *in situ*	44.4°	1.53 × 10^–3^

^a^Older regions: highland and Pre-Nectarian mares; younger regions: Mare Imbrium and Mare Orientale [5].

For the topmost regolith at the CE-4 landing site, the conductive component of the thermal conductivity has the same order as previous results [2,5,30]. Earlier work also suggests that the thermal conductivity of the topmost regolith tends to increase with the age of the lunar surface unit, which probably relates to the smaller grains in the older regions [5]. The conductive component on the surface of the CE-4 landing site is similar to the value in the older regions, i.e. ∼0.9–1.2 × 10^−3^ W m^–1^ K^–1^ [5], which agrees well with the old age of the local regolith (∼3.6 Ga) [17].

The conductive component at a depth of 1 m in the CE-4 landing site, 0.848 × 10^–2^ W m^–1^ K^–1^, is nearly consistent with the value 0.9–1.3 × 10^–2^ W m^–1^ K^–1^ at depths of 1–2 m, as yielded by Apollo 15 and 17 heat flux experiments [32].

## CONCLUSIONS

In this work, we evaluated the thermophysical properties and the grain size at the CE-4 landing site based on the local temperature probing experiment. The estimation suggests ∼15 μm for the average grain size at all depths. This value is abnormally greater than the number-weighted grain size of CE-5 samples, which were collected from a surface with a young age (∼2.0 Ga). Accounting for the old age of the CE-4 landing site (∼3.6 Ga), the estimated grain size may imply an immature regolith layer below the lunar surface. The conductive component of the thermal conductivity of the local regolith is ∼1.53 × 10^–3^ W m^–1^ K^–1^ on the surface and ∼8.84 × 10^–3^ W m^–1^ K^–1^ at a depth of 1 m.

## DATA AVAILABILITY

The CE-1 and CE-4 images were processed by the Ground Research and Application System of China's Lunar and Planetary Exploration Program and provided by China National Space Administration. The CE-1 global mosaic is accessible at http://planets.cug.edu.cn. The global map of Kaguya Optical Maturity (OMAT) is available from the United States Geological Survey (USGS) at the Planetary Data System's Cartography and Imaging Sciences Node (http://astrogeology.usgs.gov/pds/). Data sets generated are available from the corresponding author upon reasonable request.

## Supplementary Material

nwac175_Supplemental_Files
