# Supplementary material for: Thermophysical properties of the regolith on the lunar far side revealed by the in situ temperature probing of the Chang’E-4 mission
Source: Natl Sci Rev. 2022 Aug 26;9(11):nwac175. doi: 10.1093/nsr/nwac175 (PMC9646997; doi:10.1093/nsr/nwac175)
Supplement: nwac175_Supplemental_Files [file nwac175_supplemental_files.zip › [NSR_MS-2022-182] Supplementary_Information.docx]

# Supplementary Information

**Thermophysical properties of the regolith on the lunar farside revealed by the in-situ temperature probing of Chang’E-4 mission**

Xiao Xiao (肖潇)1**†**, Shuoran Yu (俞硕然)2**†**, Jun Huang (黄俊)1*, He Zhang (张熇)3, Youwei Zhang (张有为) 3, Long Xiao (肖龙)1

1 State Key Laboratory of Geological Processes and Mineral Resources, School of Earth Sciences, Planetary Science Institute, China University of Geosciences, Wuhan 430074, China.

2 State Key Laboratory of Lunar and Planetary Sciences, Macau University of Science and Technology, Macau SAR, China

3 China Academy of Space Technology, Beijing, 100094, China

* Corresponding author: Jun Huang (junhuang@cug.edu.cn)

**†** The authors contribute equally.

**Supplementary Methods**

**1. Solar position**

We use the JPL DE405 ephemeris and *lunar-sky* package [1] to determine the solar zenith angle and solar azimuth angle at the CE-4 landing site.

The solar incident angle depends not only on the solar position but also the local topography [2], i.e.

where *i* is the solar incident angle, *θp* is the slope angle, is the slope aspect, *Zs* is the solar zenith angle, *As* is the solar azimuth angle (Supplementary Figure 1). The local slope angle and slope aspect angle are 4° and 252.3°, respectively [3]. Considering the spatial resolution of the topography, we adjust the slope angle of the probes (1.15°) to get the best fit of the temperature measurements (Figure 2a).


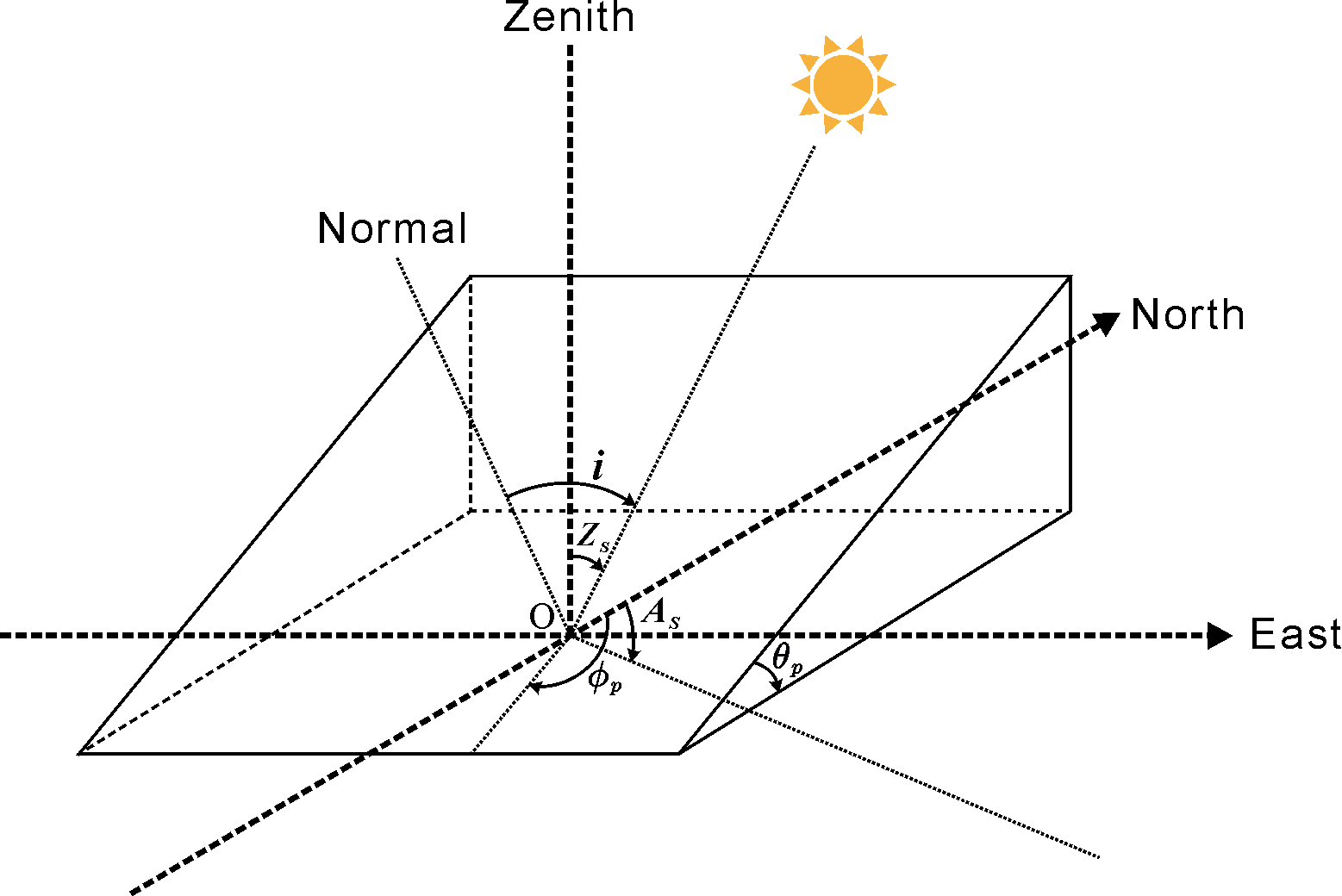


**Supplementary Figure 1.** The geometric relation between the Sun and the local topography. *i* is the solar incident angle which is the angle between the normal of the slope and the Sun, *θp* is the slope angle which is the angle between the slope and the horizontal plane, is the slope aspect which is the angle between the orientation of the slope and the north direction, *Zs* is the solar zenith angle which is the angle between the normal of the horizontal plane and the Sun, *As* is the solar azimuth angle which is the angle between the Sun and the north direction.

**2. Thermal equilibrium on the lunar surface**

In the daytime, the thermal radiation emitted from the lunar surface is balanced by the external radiation absorbed by the lunar surface, i.e.

where is the emissivity of the lunar surface (~0*.*98 following the Diviner observations [4]), *σ* is the Stefan-Boltzmann constant, *Ts* is the surface temperature, *Frad* is the external radiation flux absorbed by the lunar surface. If the lunar surface is exposed directly towards the sunshine, the external radiation flux is given by

where *I*0 is the solar radiation power, *R* is the Sun-Moon distance, *Aθ* is the albedo of the lunar surface [5]

where *A*0 is the normalized broad-band albedo (0.07 for mare and 0.12 for highland). In the context of this work, the external radiation flowing into the lunar regolith can be affected by the shadow. As the measured temperature represents the thermal condition on the lunar surface, according to the principle of thermal equilibrium, the effective radiation of the regolith can be calculated approximately by the infrared radiation of the measured temperature as:

where *Ts,p* is the measured surface temperature.

**3. Temperature of the lunar regolith**

The temperature of the lunar regolith is modeled by the heat conduction equation

where *ρ* is the bulk density, *cp* is the heat capacity, *T* is the temperature, *t* is the time, *z* is the depth, *K* is the thermal conductivity. The boundary conditions on the lunar surface and at the subsurface are

where *Ts* is the surface temperature, *J*0 is the heat flux. We set the heat flux to be zero because this parameter does not affect significantly the temperature of the topmost lunar regolith.

**4. Thermophysical properties of the lunar regolith**

As the lunar regolith is porous, its bulk density can be written as

where *G* is the density of the solid grains (unit: kg m-3), is the filling factor. The density of the solid grains (unit: kg m-3) is expressed as an empirical function [6],

where and are the FeO and TiO2 contents. The filling factor is expressed as a Fermi function of pressure [7], i.e.

where and are the filling factors of random ballistic packing and random close packing measured as 0.15 and 0.64 respectively [8,9], *p* is the hydrostatic pressure, *pm* is the turnover pressure marking the transition of packing style, Δ is the transitional width measured as ~0*.*58 for the omni-directional compression [10]. Note that the values of and are two constants independent of the grain size.

The hydrostatic pressure in the lunar regolith can be constrained via

where *p*0 is the loading pressure on the lunar surface owing to the metallic rails of the lander, *g* is the gravitational acceleration on the lunar surface.

The turnover pressure corresponds to the maximum pressure that the lunar regolith can sustain without restructuring, given by

where *F* is the adhesive bonding force, *r* is the grain radius.

Based on Hertzian contact theory and photon gas theory, the thermal conductivity of lunar regolith is determined as [11]

where *K*0 is the thermal conductivity of the solid grains, *μ* is the Poisson's ratio, *E* is the Young's modulus, *F* is the intergranular adhesive force, *χ* is a structural parameter depending on the grain radius and the filling factor [12], is the emissivity of the solid grains (unity here), *e*1 is a correction factor accounting for the irregular shape of the regolith (~1*.*34). Note that in Eq. (14), the first term specifies the conductive component occurring via the intergranular contacts (denoted by *Kc*), whereas the second term specifies the radiative component occurring via the intergranular thermal radiation (denoted by *Kr*).

Adhesive bonding force is predominant in varying the transition of filling factor and thermal conductivity over depth. Here, we determine this parameter by the John-Kendall-Robert (JKR) model [13], i.e.

where *γ* is the surface energy. Early works just dated the surface energy of the silica grains at reference temperature ~300 K. In order to evaluate the temperature dependence of the surface energy, we adopt the assumption of the linear relationship between surface energy and temperature [12], and determine the surface energy by

where *T*300 is the reference temperature of 300 K, *γ*300 is the surface energy at 300 K. The structural parameter *χ* is given by [12]

where *f*1 = 5.18×10-2 and *f*2 = 5.26.

The heat capacity of the lunar regolith is determined via an empirical function of temperature [14], i.e.

Note that Eq. (18) is ubiquitously applicable for all lunar regolith samples between 90 and 350 K.

Supplementary Table 1 shows the parameter values. Here we fix the parameters for the solid grains to the typical values and just reserve the grain radius as the only free parameter to affect the thermophysical properties of the lunar regolith.

**Supplementary Table 1**. Parameters for the thermophysical properties of lunar regolith

| Parameter | Meaning | Value | Unit |
| --- | --- | --- | --- |
|  | FeO content [15] | 12.49 | wt. % |
|  | TiO2 content [16] | 2.25 | wt. % |
|  | minimum filling factor [9] | 0.15 | – |
|  | maximum filling factor [9] | 0.64 | – |
| ∆ | transitional factor [10] | 0.58 | – |
| *G* | gravitational acceleration | 1.6 | M s-2 |
| *K*0 | thermal conductivity of solid grains [17] | 2.18 | W m-1 K-1 |
| *µ* | Poisson’s ratio of solid grains [18] | 0.25 | – |
| *E* | Young’s modulus of solid grains [18] | 7.8×1010 | Pa |
|  | emissivity of solid grains | 1.0 | – |
| *γ*300 | surface energy of silica grains at 300 K [12] | 0.02 | J m-2 |

# Supplementary Discussion 1. Thermal influences of holders and metallic rails

In this work, the topmost regolith contacts with the orange polyimide holders and metallic rails. Hence, it is necessary to examine if the overlying two materials affect the heat conduction in the topmost regolith. As shown in Supplementary Figure 2, we first consider the heat conduction in two layers with different thermal conductivities. According to Fourier's law of heat conduction, the heat flux *q* through two layers can be written as

where *K*1 and *K*2 are the thermal conductivities of the respective layers, *T*1 and *T*2 are the temperatures at the upper and lower boundaries, *T'* is the temperature at the interface between the two layers, *z1* and *z2* are the thickness of the two layers, *K* is the effective thermal conductivity of the two layers. By eliminating the temperature *T'* in Eq. (20), we obtain

or

In the context of this study, the thermal conductivities of metals and polyimide are typically hundreds of W m-1 K-1 [19] and ~0.12 W m-1 K-1 [20] respectively, far greater than the thermal conductivity of lunar regolith. By taking Eq. (21) into account, the thermal conductivity of the lunar surface should be dominated by that of lunar regolith. Hence, the contact with the overlying two materials does not affect the heat conduction in the topmost regolith.


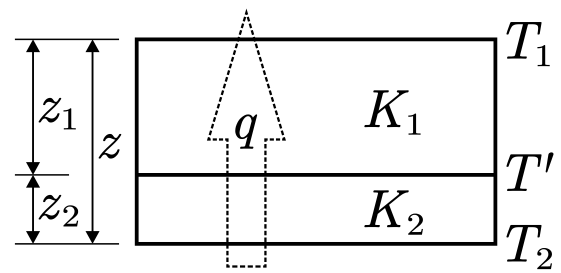


**Supplementary Figure 2.** Two contacted layers with different thermal conductivities. Here *T*1 is the temperature on the top of the overlying layer, *T*2 is the temperature at the bottom of the underlying layer, *T*’ is the temperature at the interface between two layers, *q* is the heat flux through these two layers, *z*1 and *z*2 are the thickness of the two layers, *z* is the total thickness of the two layers, *K*1 and *K*2 are the thermal conductivities of the two layers.

# Supplementary Discussion 2. Thickness of removed regolith during landing

During the landing of the CE-4 lander, the rocket exhaust can remove the topmost regolith below the nozzle. The temperature probes are installed at the end of the rail and the distance between the probes and the nozzle centerline of the CE-4 is ~5 m (Supplementary Figure 3a). According to the erosion depth modeling [21], the surface erosion depth at the position of temperature probes is only ~0.5 mm (Supplementary Figure 3b), which is nearly negligible.


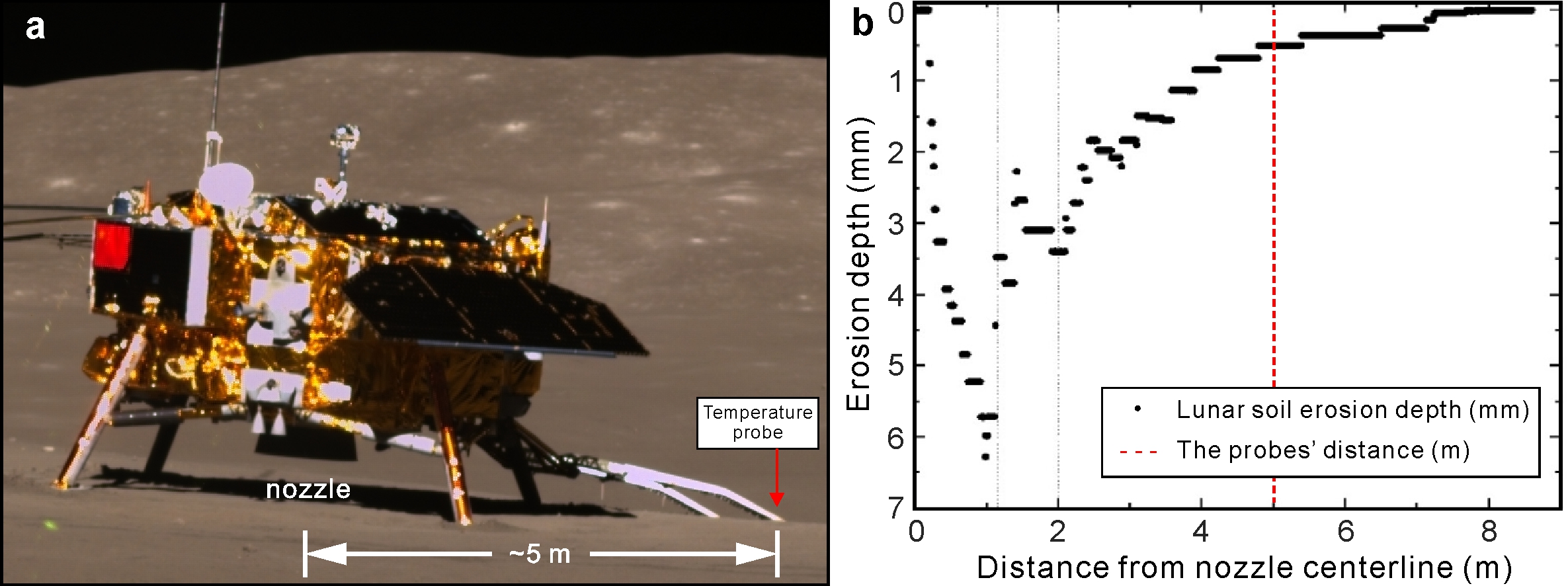


**Supplementary Figure 3.** (a) The distance (~5 m) between the temperature probes and the nozzle centerline of CE-4. The temperature probes are at the end of the rail. (b) The variation of erosion depth over the distance from the nozzle centerline obtained from the erosion modeling [21].

# Supplementary Discussion 3. Shading effect of the rails and the lander

Different from the remote sensing observations, the *in-situ* temperature probing is possibly affected by the shadow of the surrounding features. In the context of this work, the shading effect is related to the metallic rails and the lunar lander itself. To examine the shading effect of metallic rails, we consider a shading angle by projecting the slope angle of metallic rails onto the vertical plane constrained by the sunshine, i.e.

where is the shading angle in the vertical plane of the sunshine, is the slope angle of the metallic rails. If the solar altitude angle is greater than , the temperature probes fall into the shadow of the metallic rails.

Supplementary Figure 4b shows the variation of over time and its comparison with the solar altitude angle. The solar altitude angle is always greater than . Correspondingly, the temperature probes always fall into the shadow of the metallic rails.

As shown in Supplementary Figure 4a, is the angle between the local horizon and the line from the temperature probe to the top of the lunar lander. Near the lunar noon, the temperature probes fall into the shadow of the lunar lander as the solar altitude angle (the maximum is 44.2°) is less than (~49°).


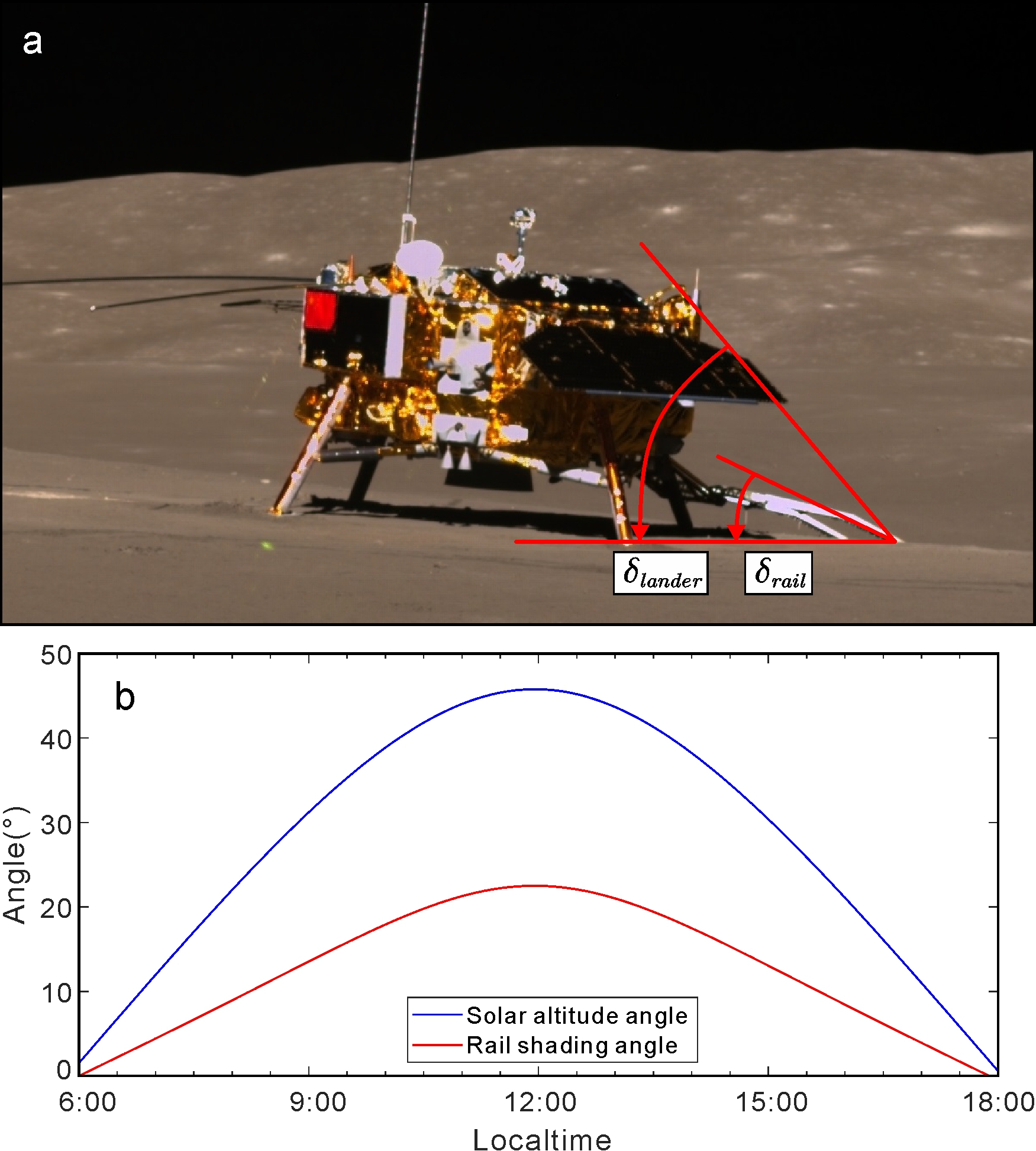


**Supplementary Figure 4.** The shading effect of the metallic rails and the lander. (a) The measurements for the slope angle of the metallic rails (*δrail*) and the angle between the local horizon and the line from the temperature probes to the top of the lunar lander (*δlander*). (b) The comparison between the solar altitude angle and the shading angle of the metallic rails.

# Supplementary Discussion 4. Radiative Cooling of metallic rails in the nighttime

In the nighttime, the thermal radiation of the metallic rail may slow down the cooling of the probed regolith. Here we prove with a simple model that this effect is nearly negligible. Assuming the thin metallic panel to be isothermal, we obtain the following energy conservation equation

where *ρ* is the density, *cp* is the heat capacity, *V* is the volume, *A* is the area of the panel. By integrating Eq. (23), we obtain

or

where *T*b is the temperature at the beginning of cooling, *T*e is the temperature at the end of cooling, *z* is the thickness of the panel, is the cooling time. It is hard to calculate the temperature of metallic rails at the time of sunset, i.e., the beginning of cooling. Alternatively, we assume *Tb3 >> Te3* and the cooling time can be approximated as

Note that the assumption *Tb3 >> Te3* can over-estimate the cooling time.

In the nighttime, the temperature of the probed regolith is maximal ~100 K. Besides, the metallic rails are made of aluminum alloy panel with a thickness of ~1 mm. Given *ρ* = ~2700 kg m-3, *cp* = ~890 J kg-1 K-1, *z* = ~0.001 m, = ~1.0, the thermal radiation flux from metallic rails tends to be less than 10 % of the thermal radiation flux from the probed regolith after ~0.4 lunar hours (i.e., ~11 terrestrial hours). Accounting for the over-estimation of the cooling time, the thermal radiation from metallic rails can be neglected throughout the whole nighttime.

# Supplementary Discussion 5. Grain size estimation at the Chang’E-5 landing site

We investigate the grain size at the Chang’E-5 (CE-5) landing site by the Diviner bolometric temperature data at 43.25°N, 51.75°W, which represent the average condition an area (0.5° in width) [22] containing the CE-5 landing site (43.06°N, 51.92°W) [23]. The fitting results are presented in Supplementary Figure 5.


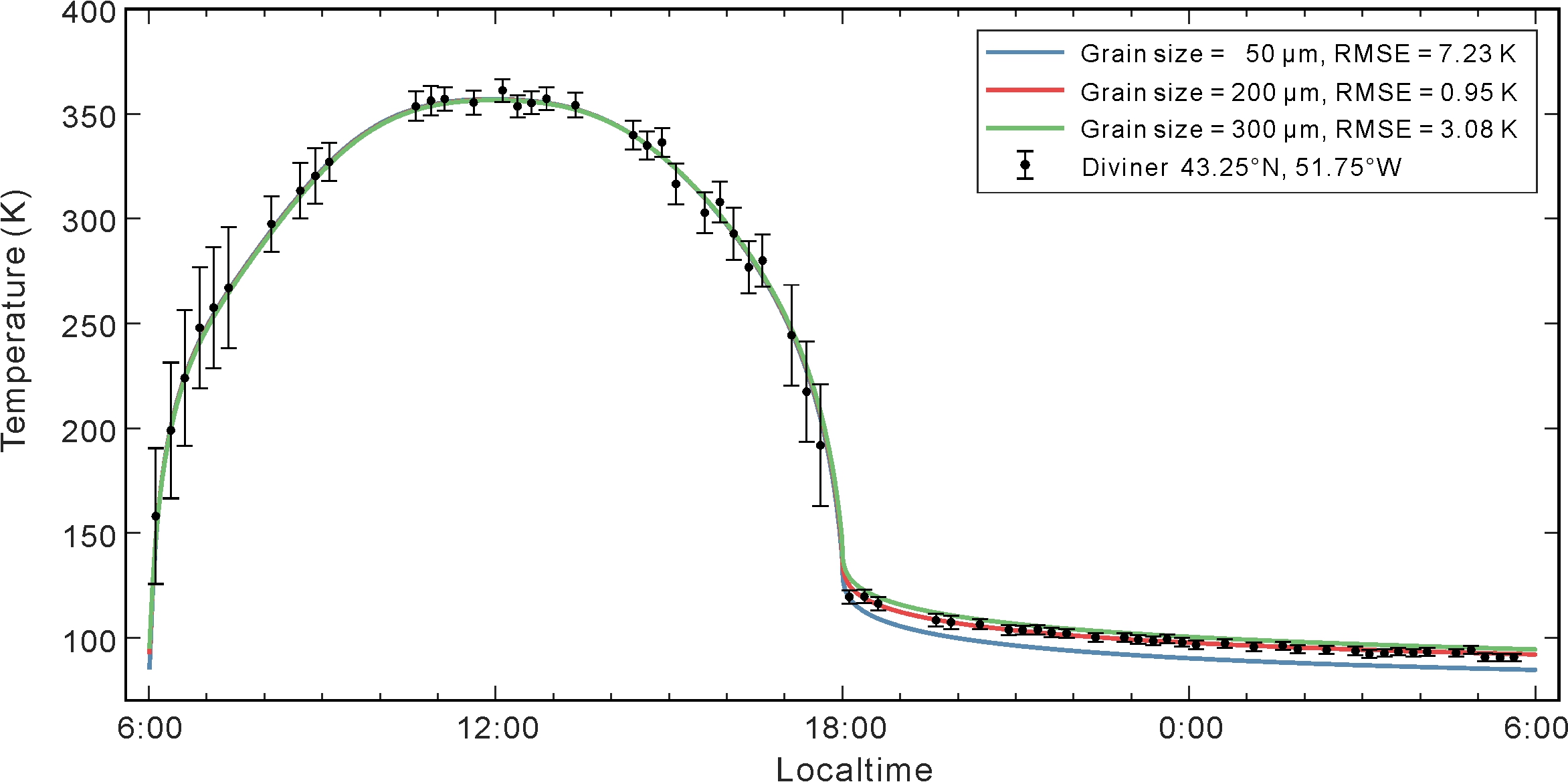


**Supplementary Figure 5.** The fitting results based on the Diviner bolometric temperature for an area (43.25°N, 51.75°W, 0.5° in width) [22] containing the CE-5 landing site (43.06°N,51.92°W) [23]. The black dots specify the bolometric temperature, and the black bars specify the errors. The colored curves represent the surface temperatures for different grain sizes. The best fit between modeled surface temperature and bolometric temperature is achieved with a grain size of ~200 μm, or 110-330 μm accounting for the errors.


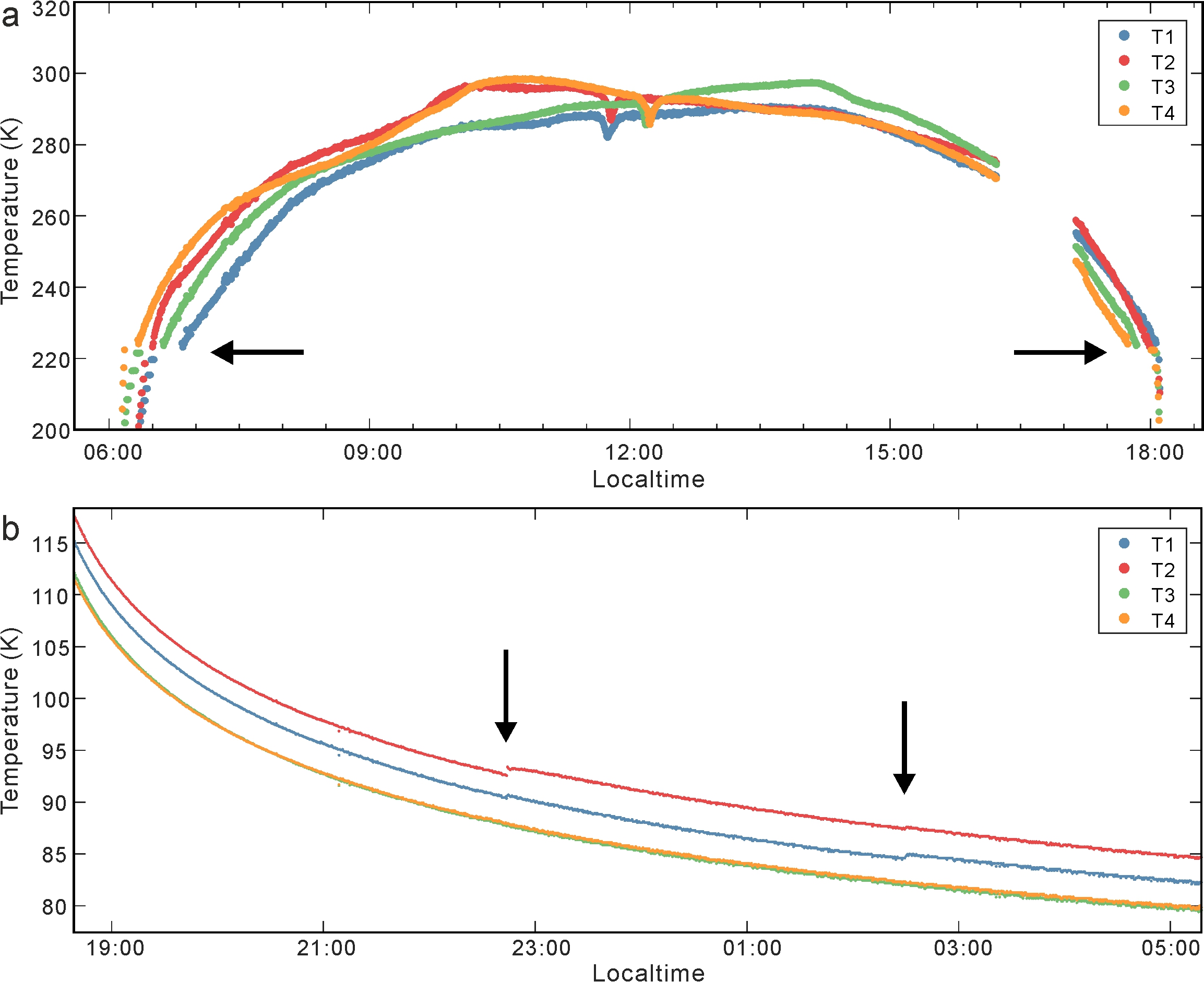


**Supplementary Figure 6.** The temperature variation of the probes at the CE-4 landing site. (a) The temperature variation obtained during the daytime The temperatures of the probes are discontinuous in time at the threshold temperature (~223 K) (black arrows). (b) The temperature variation during the fourth lunar night. The temperatures of T1 and T2 are discontinuous as the black arrows indicate.


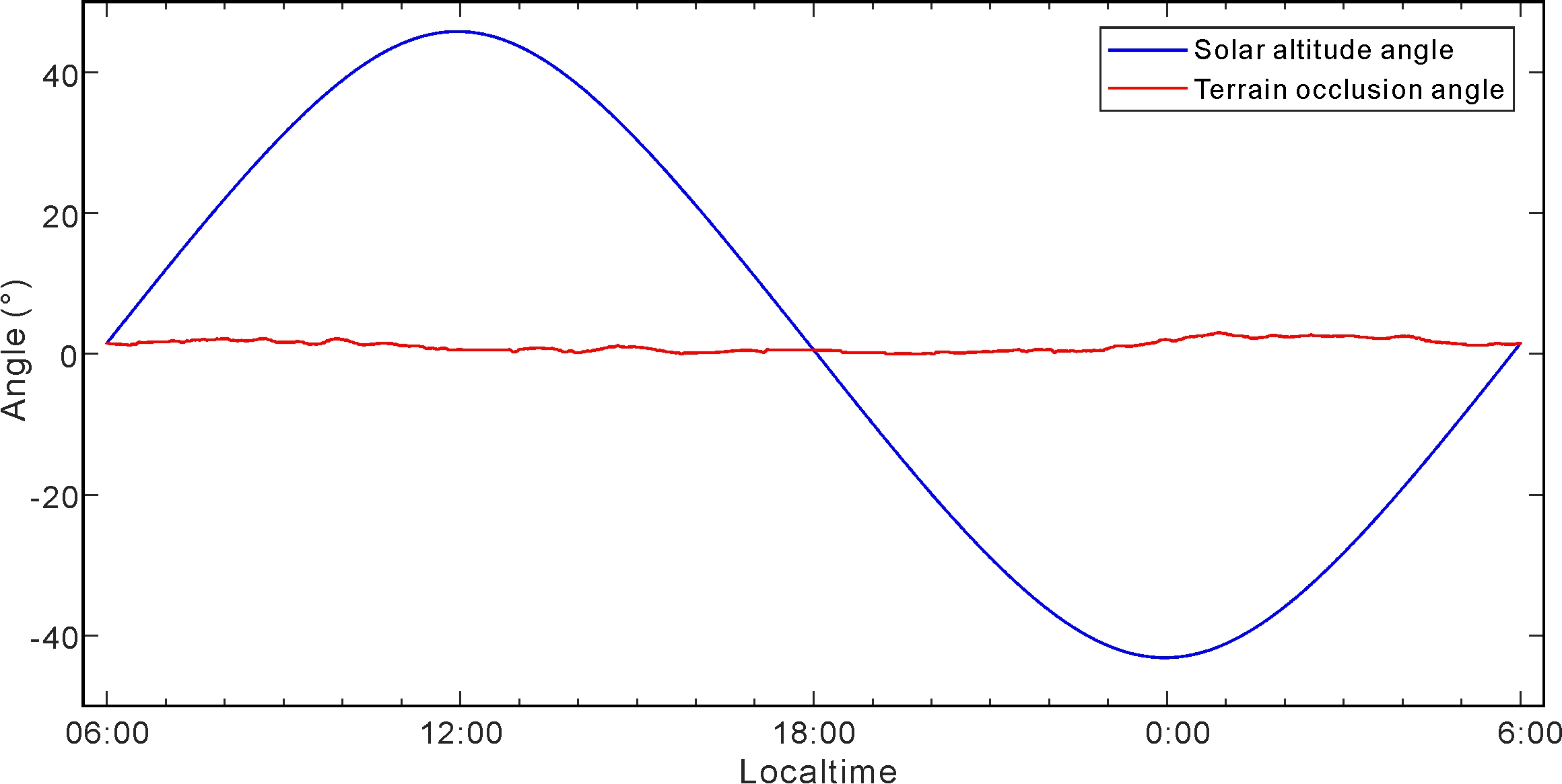


**Supplementary Figure 7.** The time variation of solar altitude angle and terrain occlusion angle [24] at the CE-4 landing site. Sunrise and sunset occur when these two angles are identical.


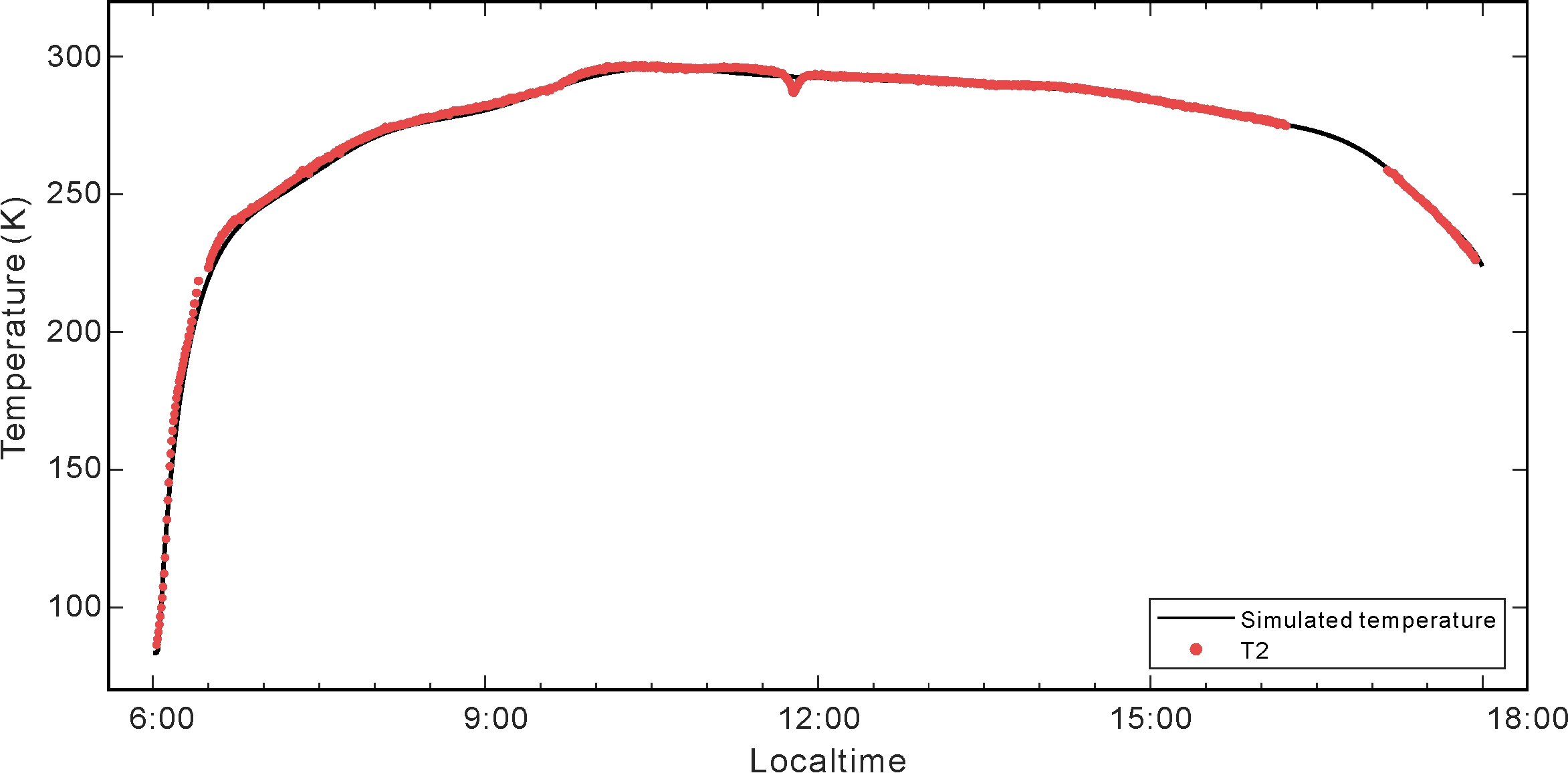


**Supplementary Figure 8.** Comparison between simulated temperatures and measured temperatures during the daytime. The black line represents the daytime temperatures derived from the effective radiation flux (see Supplementary Methods). The red scatters represent the daytime temperatures probed by the CE-4 lander's temperature sensors.


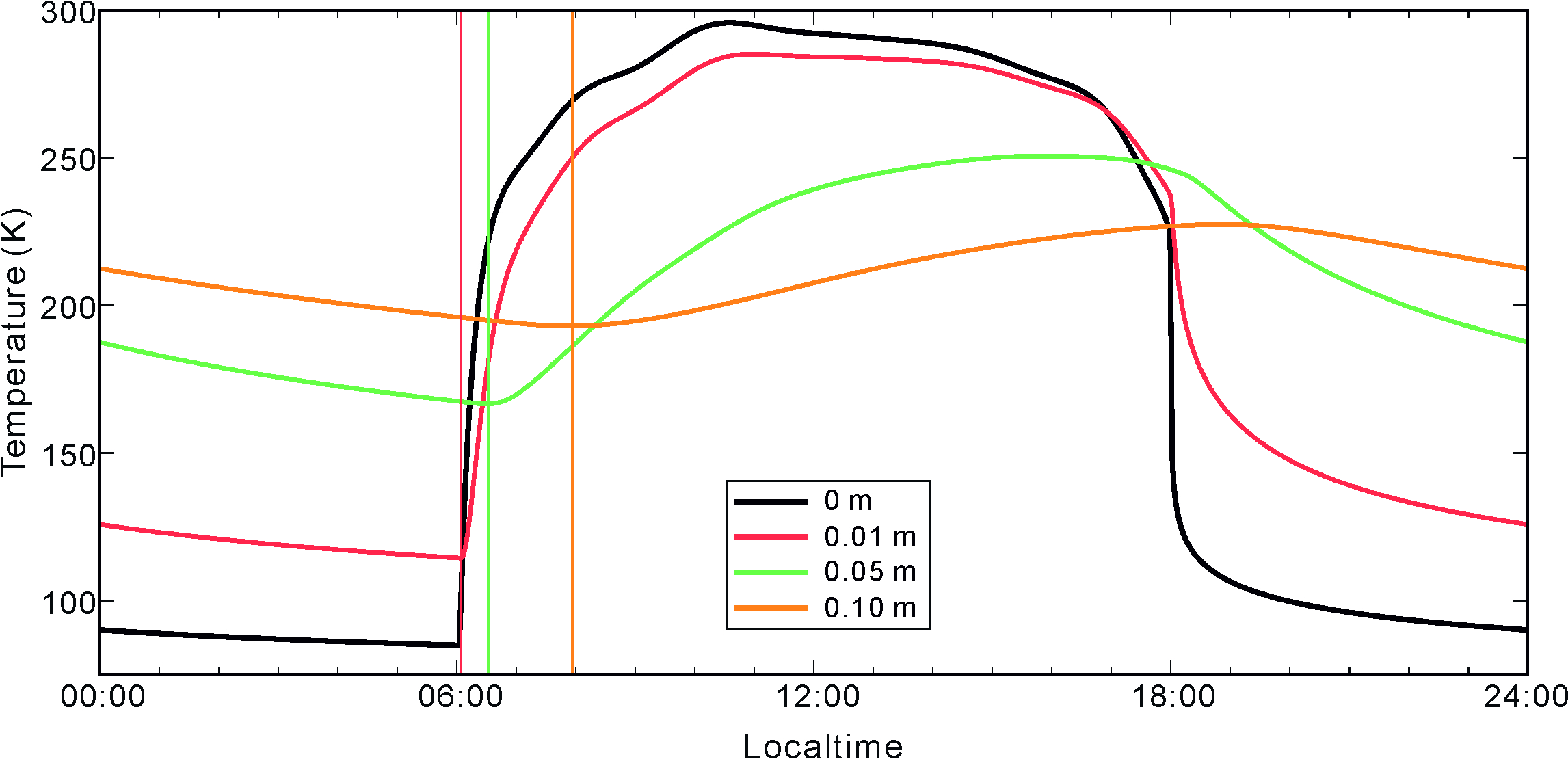


**Supplementary Figure 9.** The modeled temperatures of the lunar regolith for different depths at the CE-4 landing site. The vertical lines indicate the time at which the temperature increase begins.

**Supplementary Table 2.** Temperature variation with respect to the last measurement at different time for all four temperature probes

| Time | Temperature variation (K) | | | |
| --- | --- | --- | --- | --- |
|  | T1 | T2 | T3 | T4 |
| 2019/2/27 8:43 | -0.2 | -0.1 | -0.2 | -0.2 |
| 2019/2/27 8:58 | 0.2 | 0.1 | 0.2 | 0.3 |
| 2019/2/27 9:13* | -0.2 | 0.0 | -0.2 | -0.2 |
| 2019/2/27 9:28 | 0.5 | 0.4 | 0.7 | 0.7 |
| 2019/2/27 9:43 | 0.5 | 0.4 | 0.9 | 0.9 |
| 2019/2/27 9:58 | 0.9 | 1.1 | 1.9 | 1.9 |

*After the time, these temperatures of all probes begin to increase. The positive increment indicates the case of sunrise.

# Supplementary References

1. Ye HL, Guo HD and Liu G *et al.* Looking Vector Direction Analysis for the Moon-Based Earth Observation Optical Sensor. *IEEE J Sel Top Appl Earth Obs Remote Sens* 2018; **11**: 4488–99.

2. Yu SR and Fa WZ. Thermal conductivity of surficial lunar regolith estimated from Lunar Reconnaissance Orbiter Diviner Radiometer data. *Planet Space Sci* 2016; **124**: 48–61.

3. Wu B, Li Y and Liu WC *et al.* Centimeter-resolution topographic modeling and fine-scale analysis of craters and rocks at the Chang’E-4 landing site. *Earth Planet Sci Lett* 2021; **553**: 116666.

4. Vasavada AR, Bandfield JL and Greenhagen BT *et al.* Lunar equatorial surface temperatures and regolith properties from the diviner lunar radiometer experiment. *J Geophys Res Planets* 2012; **117**: E00H18.

5. Feng JQ, Siegler MA and Hayne PO. New Constraints on Thermal and Dielectric Properties of Lunar Regolith from LRO Diviner and CE‐2 Microwave Radiometer. *J Geophys Res Planets* 2020;**125**: e2019JE006130.

6. Huang Q and Wieczorek MA. Density and porosity of the lunar crust from gravity and topography. *J Geophys Res Planets* 2012; **117**: E05003.

7. Schräpler R, Blum J and von Borstel I *et al.* The stratification of regolith on celestial objects. *Icarus* 2015; **257**: 33–46.

8. Yang RY, Zou RP and Yu AB. Computer simulation of the packing of fine particles. *Phys Rev E* 2000; **62**: 3900–8.

9. Blum J, Schräpler R. Structure and Mechanical Properties of High-Porosity Macroscopic Agglomerates Formed by Random Ballistic Deposition. *Phys Rev Lett* 2004; **93**: 115503.

10. Güttler C, Krause M and Geretshauser RJ *et al.* The Physics of Protoplanetesimal Dust Agglomerates. IV. Toward a Dynamical Collision Model. *Astrophys J* 2009; **701**: 130–41.

11. Gundlach B and Blum J. A new method to determine the grain size of planetary regolith. *Icarus* 2013; **223**: 479–92.

12. Gundlach B and Blum J. Outgassing of icy bodies in the Solar System - II. Heat transport in dry, porous surface dust layers. *Icarus* 2012; **219**: 618–29.

13. Johnson KL, Kendall K and Roberts AD. Surface energy and the contact of elastic solids. *Proc R Soc Lond A* 1971; **324**: 301–13.

14. Keihm SJ. Interpretation of the lunar microwave brightness temperature spectrum: Feasibility of orbital heat flow mapping. *Icarus* 1984; **60**: 568–89.

15. Lemelin M, Lucey PG and Gaddis LR *et al.* Global Map Products from the Kaguya Multiband Imager at 512 ppd: Minerals, FeO, and OMAT. Abstract 2994, 47th Lunar and Planetary Science Conference, Woodlands TX, 21–25 March 2016.

16. Sato H, Robinson MS and Lawrence SJ *et al.* Lunar mare TiO2 abundances estimated from UV/Vis reflectance. *Icarus* 2017; **296**: 216–38.

17. Opeil CP, Consolmagno GJ and Britt DT. The thermal conductivity of meteorites: New measurements and analysis. *Icarus* 2010; **208**: 449–54.

18. Schultz RA. Limits on strength and deformation properties of jointed basaltic rock masses. *Rock Mech Rock Engng* 1995; **28**: 1–15.

19. Uher C. Thermal Conductivity of Metals. In: Tritt TM (ed.). *Thermal Conductivity*. Springer, Boston, MA, 2004, 21–91.

20. Dong L, Xi Q and Chen DS *et al.* Dimensional crossover of heat conduction in amorphous polyimide nanofibers. *Natl Sci Rev* 2018; **5**: 500–6.

21. You JL, Zhang XP and Zhang HY *et al.* Analysis of plume–lunar surface interaction and soil erosion during the Chang’E-4 landing process. *Acta Astronaut* 2021; **185**: 337–51.

22. Williams J-P, Paige DA and Greenhagen BT *et al.* The global surface temperatures of the Moon as measured by the Diviner Lunar Radiometer Experiment. *Icarus* 2017; **283**: 300–25.

23. Wang J, Zhang Y and Di KC *et al.* Localization of the Chang’e-5 Lander Using Radio-Tracking and Image-Based Methods. *Remote Sens* 2021; **13**: 590.

24. Wu B, Li F and Hu H *et al.* Topographic and Geomorphological Mapping and Analysis of the Chang’E-4 Landing Site on the Far Side of the Moon. *Photogramm Eng Remote Sens* 2020; **86**: 247-58.
